# Design of a Magnesium Microstructured Biohybrid Material for Practical Atmospheric CO_2_ Mitigation

**DOI:** 10.1021/acsaem.5c03841

**Published:** 2026-02-10

**Authors:** Carla Garcia-Sanz, Jose M. Palomo

**Affiliations:** Instituto de Catálisis y Petroleoquímica (ICP), CSIC, c/Marie Curie 2, Madrid 28049, Spain

**Keywords:** microstructured biohybrid, CO_2_ transformation, catalytic material, gas-phase reaction, heterogeneous catalysis

## Abstract

The rising levels of greenhouse gases such as CO_2_ pose critical challenges for climate stability and indoor air quality. Here, we report the design and synthesis of a magnesium-based microstructured biohybrid (MicroMg) using a mild, enzyme-assisted process at room temperature and neutral pH. MicroMg consists of well-defined Mg_3_(PO_4_)_2_ microstructures stabilized by a lipase scaffold, exhibiting high structural integrity and crystallinity. In aqueous media, MicroMg efficiently converts CO_2_ into mainly bicarbonate under ambient conditions, achieving complete conversion of aqueous CO_2_ within 30 min (TOF value of 16 h^–1^) and demonstrating structural stability over repeated reactions. When this was incorporated into paint and applied to real wall surfaces, MicroMg effectively reduced CO_2_ concentrations in gas-phase experiments, maintaining >90% of the initial activity over three washing cycles and performing better on larger coated areas (35 cm^2^) and with double-layer applications. Additionally, MicroMg remained active at elevated CO_2_ concentrations (up to 1500 ppm), with a transformation rate of 16 ppm/h of CO_2_ confirming its potential for mitigating indoor CO_2_ levels. These results demonstrate that MicroMg is a sustainable, reusable, and scalable material for the CO_2_ transformation, offering a promising strategy for both indoor air quality improvement and greenhouse gas mitigation.

## Graphical abstract

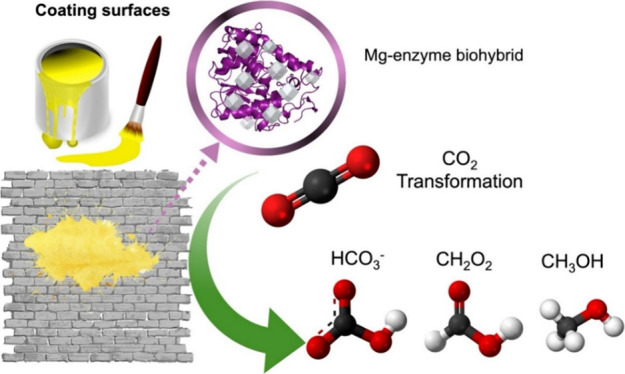

## Introduction

Current projections for greenhouse gas emissions and global warming suggest that negative-emission technologies capable of actively removing CO_2_, methane, or nitrous oxide from the atmosphere will likely become essential to ensure a stable climate for future generations.^1–3^ Recently, the United Nations Climate Change Conference (COP29, Azerbaijan, November 2024) reinforced global awareness of the urgent actions required to curb global warming. The conference also reaffirmed a framework for climate change mitigation aligned with the United Nations Sustainable Development Goals (SDGs), particularly SDG 13–Climate Action, while also supporting SDG 11–Sustainable Cities and Communities through improved indoor air quality and healthier living environments and promoting SDG 9–Industry, Innovation, and Infrastructure by fostering scalable, low-energy, and efficient technological solutions.^4^ Furthermore, the Intergovernmental Panel on Climate Change (IPCC) set the target of keeping warming below 2 °C above preindustrial levels, while pursuing efforts to limit the temperature increase to 1.5 °C.^5^ Thus, a substantial and practical shift in the way we capture and transform pollutant gases such as CO_2_, methane, and NO_*x*_—with high capacity, rational regeneration, and low energy penalty—is urgently needed.

While CO_2_ capture for underground storage has been discussed for decades, engineering challenges and concerns about potential leaks have hindered implementation.^6,7^ Alternatively, the chemical conversion of CO_2_ into nonvolatile products could provide a permanent storage solution. Ideally, reducing CO_2_ or methane to value-added chemicals—usable either as fuels or as feedstocks for the chemical industry through renewable energy sources—represents a crucial pathway toward a carbon-neutral future.^8–10^

At the same time, CO_2_ accumulation is a growing concern indoors, where people in industrialized nations spend nearly 90% of their time.^11,12^ Indoor concentrations can rise far above outdoor levels, particularly in energy-efficient buildings with reduced ventilation. Good indoor air quality is typically associated with CO_2_ levels below 600–700 ppm, while concentrations up to 1000 ppm are considered acceptable.^13^ Exceeding 1000 ppm indicates insufficient ventilation and may cause drowsiness, reduced cognitive performance, and general discomfort.^13,14^ Because CO_2_ levels closely reflect human occupancy and ventilation efficiency, they are widely used as a proxy for indoor air quality. Given that the global outdoor average reached approximately 422 ppm in 2024, developing innovative strategies to actively reduce CO_2_ both indoors and outdoors has become an urgent priority.^15^

In this context, nanotechnology provides a broad range of capabilities, particularly through nanomaterials and metal nanoparticle (NP) catalysts, which enable the efficient transformation of pollutant gases under mild conditions.^16,17^ Their very high surface area makes them excellent catalysts, requiring less material per gram of product and thus improving sustainability. Nevertheless, conventional chemical synthesis of NPs often involves hazardous conditions, toxic solvents, or high energy input, which hinder large-scale applications. To address these limitations, recent strategies have exploited biomolecules, such as enzymes, to induce the *in situ* formation of metal nanoparticles, controlling their size and shape while avoiding aggregation.^18–20^ These enzyme–nanoparticle biohybrids represent a new class of eco-friendly nanocatalysts for the transformation of greenhouse gases.^21^

Magnesium-based catalysts have emerged as being particularly attractive for these applications. In the literature, Mg-based materials have been reported for organic transformations, hydrogen generation, and thermal and electrical energy production.^22–25^ However, few studies have examined their interactions with CO_2_.^26–28^ Magnesium is the eighth most abundant element in the Earth’s crust and the fourth most common element on the planet (after oxygen, silicon, and iron).^29^ Its high natural abundance and extremely low cost—between 45,000–50,000 times cheaper than other metallic catalysts traditionally used for CO_2_ reduction—make it highly suitable for large-scale applications. Moreover, magnesium and its oxides react efficiently with CO_2_ under mild conditions, enabling the formation of value-added products such as bicarbonate, formic acid, and methanol.^30–32^ Magnesium also integrates well into enzyme-based biohybrid systems, allowing the formation of stable, well-dispersed nanoparticles while minimizing aggregation. In addition, as a nontoxic and environmentally friendly metal, magnesium supports sustainable catalytic strategies compared to heavy metal alternatives.^33^

Therefore, in this work, we have designed a new type of magnesium-based biohybrid that reacts directly with CO_2_ (from air or water) under ambient temperature and pressure, producing bicarbonate, formic acid, and methanol without the need for external energy sources. Importantly, this system has been applied to coat real wall surfaces, demonstrating the potential to reduce the CO_2_ concentrations in enclosed spaces (Figure 1). This approach provides a promising strategy for both sustainable greenhouse gas mitigation and the improvement of indoor air quality.

**Figure 1:**
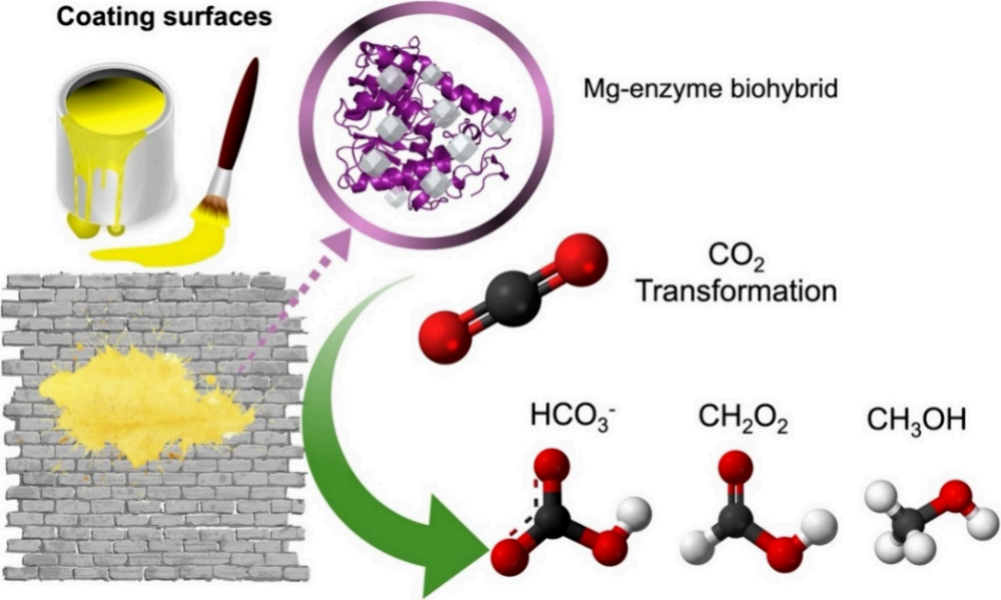
Schematic representation of the surface-coated magnesium micromaterial proposed in this work for the CO_2_ transformation.

## Experimental Section

### Materials

Sodium dihydrogen phosphate dihydrate (NaH_2_PO_4_·2H_2_O, CAS: 7558-79-4) and sodium hydroxide (NaOH, CAS: 1310-73-2) were purchased from Labkem (Barcelona, Spain). Magnesium sulfate heptahydrate (MgSO_4_·7H_2_O, CAS: 10034-99-8) was obtained from Sigma-Aldrich (MA, USA). Lipase B from *Candida antarctica* (CALB) (Lipozyme CalB) was supplied by Novonesis (formerly Novozymes) (Bagsværd, Denmark). Liquid nitrogen (N_2_) and carbon dioxide (CO_2_) were supplied by Air Liquide (Paris, France). Paint (slight yellow, batch no. 01212227) was kindly provided by Decamed Trading S.L.

### Characterization and Analytical Methods

Spectrophotometric analyses were carried out using a V-730 spectrophotometer (JASCO, Tokyo, Japan). Infrared spectra of the Mg nanomaterials were recorded on an FT/IR-4600 spectrophotometer (JASCO, Tokyo, Japan). Inductively coupled plasma-optical emission spectroscopy (ICP-OES) was employed to determine the elemental composition of the solid materials. For this, 5 mg of solid powder was digested with 6 mL of HCl (37% v/v), followed by the addition of 9 mL of water. The resulting solution was centrifuged, and the clear supernatant was analyzed for magnesium content using an OPTIMA 2100 DV instrument (PerkinElmer, Waltham, MA, USA). X-ray diffraction (XRD) patterns were collected using a PANalytical X’Pert Pro diffractometer with a D8 Advance analysis texture configuration (Bruker, Billerica, MA) and Cu Kα radiation (λ = 1.5406 Å, 45 kV, 40 mA). Data analysis was performed using X’Pert Data Viewer and X’Pert Highscore Plus software. The size and morphology of the Mg-based micromaterial were examined by scanning electron microscopy (SEM) using a TM-1000 microscope (Hitachi, Tokyo, Japan). Samples were prepared by depositing a small amount of the material onto a thin, conductive carbon-coated film. SEM images were acquired using an electron beam acceleration voltage of 15 kV, with a working distance of approximately 8–10 mm, operating in high-vacuum mode and using the secondary electron detector. Image acquisition was performed under standard lens conditions optimized for surface morphology observation. Transmission electron microscopy (TEM) was also employed to determine the particle size and morphology. TEM imaging was performed using an S/TEM Titan 80–300 microscope equipped with a CETCOR Cs probe corrector and an energy-dispersive X-ray spectrometer (EDS) for chemical composition analysis. For TEM sample preparation, a small amount of material was dispersed in ethanol and a droplet of the suspension was placed onto a copper grid coated with a carbon film. The solvent was allowed to evaporate, after which the samples were dried and plasma-cleaned. Both TEM (bright field, dark field, and selected-area diffraction) and STEM modes (BF for structure and morphology; HAADF for chemical contrast and *Z*-contrast) were used. Due to the beam sensitivity of the samples, electron beam intensity and exposure times were minimized during imaging. CO_2_ reactions were conducted in both liquid and gas phases. Liquid-phase reactions were analyzed by high-performance liquid chromatography (HPLC) using a PU-4180 pump and a UV-4075 detector (JASCO, Tokyo, Japan) at 25 °C, while gas-phase reactions were monitored with a carbon dioxide sensor (model AZ 7530, AZ Instrument Corp., Taiwan) covering a sensitivity range of 0–5000 ppm.

### General Synthesis of the Mg–Enzyme Biohybrid (MicroMg)

For the preparation of the Mg–enzyme biohybrid, 1.6 mL of commercial CALB solution (10.36 mg/mL, determined by Bradford assay) was diluted in 60 mL of 0.1 M sodium phosphate buffer (pH 7), resulting in a final enzyme concentration of 0.3 mg/mL. The solution was transferred to a 100 mL glass bottle containing a small magnetic stir bar. Subsequently, magnesium sulfate heptahydrate (MgSO_4_·7H_2_O, 600 mg, 10 mg/mL) was added, and the mixture was magnetically stirred at room temperature for 17 h using a 0.5 × 1.5 cm stir bar at 400 rpm. After incubation, the suspension was centrifuged at 8000 rpm for 10 min, and the resulting pellet was washed three times with distilled water (3 × 10 mL). The washed solids were resuspended in 2 mL of water, transferred to cryotubes, frozen in liquid nitrogen, and lyophilized overnight. The final micromaterial was obtained as a white powder (0.253 g) and was designated as MicroMg.

### CO_2_ Liquid-Phase Transformation Reaction

For the CO_2_ transformation, 5 mL of an aqueous solution saturated with 314 ppm CO_2_ was mixed with 20 mg of **MicroMg**. The reaction was carried out for 1 min to 2 h at room temperature under constant stirring and natural light. Conversion of CO_2_ was determined by HPLC. Bicarbonate and formic acid were analyzed using a Phenomenex Gemini-NX C18 column (250 × 4.6 mm, 5 μm) with a mobile phase of H_2_O Milli-Q/ACN/TFA (90:10, pH 4.3) at a flow rate of 1 mL/min. Detection was performed with a UV detector at 210 nm. Samples were diluted 1:1 with the mobile phase prior to injection. Under these conditions, the retention times were 2.3 min for bicarbonate and 4.1 min for formic acid. Methanol analysis was performed on a Phenomenex Amino LUNA column (250 × 4.6 mm, 5 μm) at 30 °C with a mobile phase of Milli-Q water adjusted to pH 5 with 8 mM H_2_SO_4_, at a flow rate of 1 mL/min. Methanol was detected using a refractive index (RI) detector with a retention time of 3.9 min. Conversions of bicarbonate, formic acid, and methanol were calculated from calibration curves obtained with standards at different concentrations. The TON (mmol CO_2_/mmol %Mg in MicroMg) value was calculated at a conversion of around 50%. The TOF value was calculated using this equation: TOF (h^–1^) = TON/time (h).

### MicroMg-Paint Coating on Real Wall Surfaces

The reaction was further tested on real wall samples. MicroMg (0.4 g) was dissolved in 100 mL of distilled water, obtaining an emulsion mixture of 4000 ppm as stock. Then, different paint formulations were prepared as described in Table 1, by adding different amounts of MicroMg solution to the paint, resulting in a final MicroMg concentration from 174 to 700 ppm.

**Table 1:** Composition of Paint Coatings Containing MicroMg

sample	MicroMg (mL)	paint (mL)	water (mL)	concentration of MicroMg (ppm)
1	0.25	4.75	0.25	174
2	0.5	4.5	0.5	350
3	1	4	1	700

Then, each formulation was manually applied with a brush onto wall sections of approximately 6 × 4 cm^2^ (24 cm^2^) in a single layer. The coated sections were then left to dry at room temperature for 24 h, after which the surfaces were characterized by scanning electron microscopy (SEM) to evaluate the morphology and uniformity.

### CO_2_ Gas-Phase Transformation Reaction

Wall sections of 24 cm^2^ coated with varying concentrations of MicroMg were placed in a sealed plastic chamber equipped with a CO_2_ sensor. CO_2_ was introduced until the concentration reached 800–900 ppm, and the reaction was monitored over 24 h, with CO_2_ levels recorded continuously to calculate conversion relative to the initial concentration. The same experiment was then repeated using a larger wall section of 35 cm^2^. Experiments were subsequently performed at higher CO_2_ concentrations, up to 1500 ppm, and transformations were followed for over 72 h.

### Washing Cycle Experiments of the Coated Wall Surfaces

The efficiency of the nanomaterial was evaluated by multiple washing cycles. Coated wall samples were washed with 5 mL of distilled water and then allowed to dry at room temperature. Subsequently, the gas-phase CO_2_ reaction was repeated to assess whether the catalyst retained its activity after washing. This procedure was repeated three times, corresponding to a total of three water washes.

## Results and Discussion

### Synthesis and Characterization of MicroMg

First, the novel magnesium-based microstructured biohybrid was designed and synthesized under mild conditions, at room temperature and neutral pH, through a bioinduced process in which the enzyme interacts with the magnesium salt (Figure 2a). In the synthesis, 0.3 mg/mL of protein *Candida antarctica* lipase B (CALB, 33 kDa) was incubated with MgSO_4_ (10 mg/mL) for 17 h at room temperature. The resulting solid was collected by centrifugation, frozen, and lyophilized to yield the final hybrid, designated **MicroMg**. The metallic species in **MicroMg** was determined by wide-angle X-ray diffraction (XRD), which revealed the characteristic peaks of Mg_3_(PO_4_)_2_ (Figure 2b). XRD analysis showed a consistent pattern, with diffraction peaks at 2θ = 18.09° ( $10\overset{-}{1}$ ), 21.07° (101), 24.46° ( $1\overset{-}{1}\overset{-}{1}$ ), 28.43° ( $02\overset{-}{2}$ ), 29.99° (211), 34.67° (220), and 40.76° ( $32\overset{-}{3}$ ), corresponding to Mg_3_(PO_4_)_2_ (JCPDS 35-0329).^34^ The presence of these species was further confirmed by Fourier transform infrared spectroscopy (FT-IR) (Figure 2c). The characteristic ν Mg–O stretching and bending vibrations were observed at 807 cm^–1^. Additionally, absorption bands at 1106 and 1071 cm^–1^ corresponded to the asymmetric and symmetric stretching of PO_4_^3–^ groups, while the δ P–O bending vibrations appeared at 630 and 566 cm^–1^.^34^ Other bands in the spectrum were attributed to the protein-associated water molecules, showing a broad band centered at 3317 cm^–1^ characteristic of −OH stretching vibrations, and to the carboxyl groups of aspartic and glutamic acid residues, with bands around 1615 and 1390 cm^–1^.^35^

**Figure 2:**
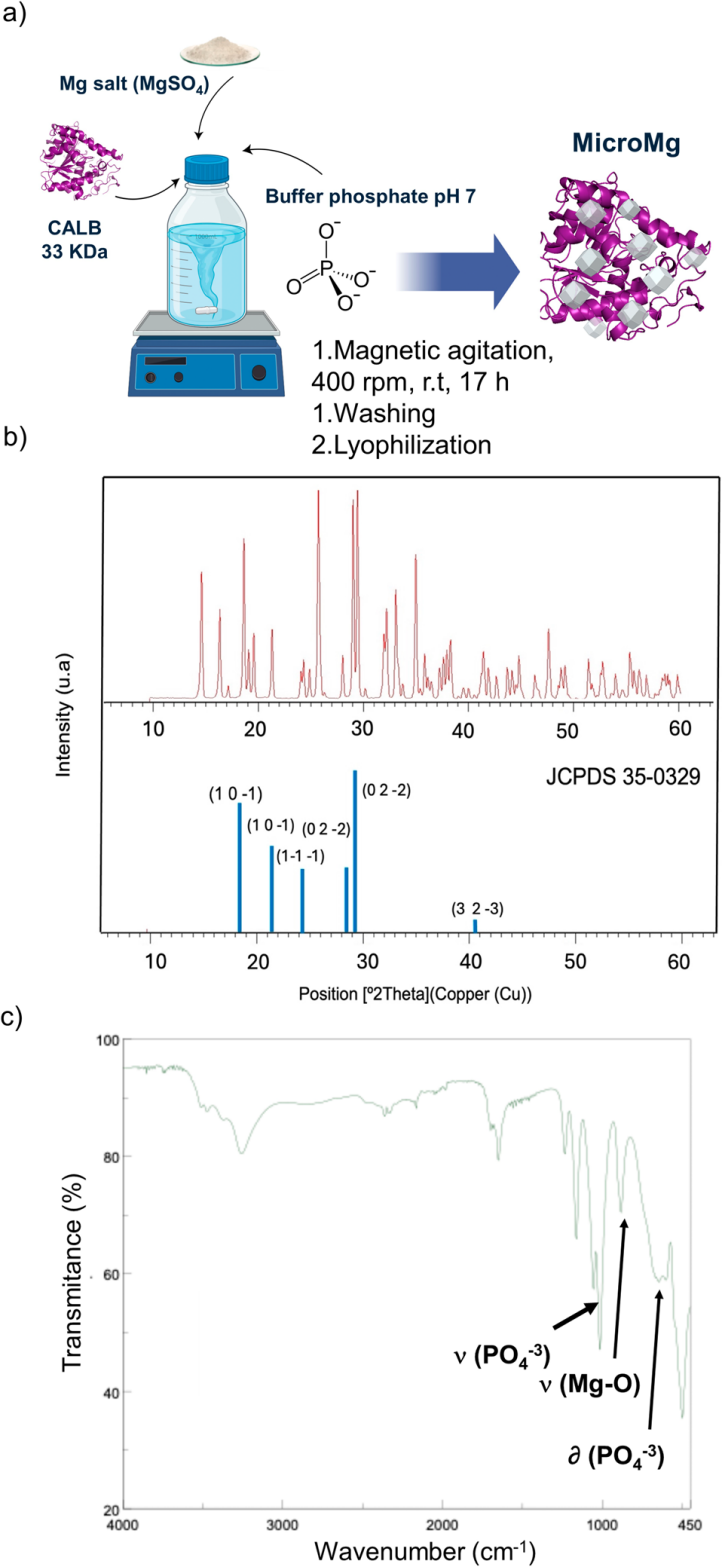
Synthesis and characterization of the MicroMg; (a) scheme of synthesis; (b) X-ray diffraction patterns; (c) FT-IR spectrum.

Finally, to confirm the exclusive formation of Mg_3_(PO_4_)_2_, X-ray photoelectron spectroscopy (XPS) was performed (Figure S1). The analysis revealed a single component in the P 2p orbital corresponding to the phosphate ion, with a binding energy of 136.4 eV, and a single component in the Mg 2p orbital corresponding to Mg^2+^, with a binding energy of 53 eV. Furthermore, the Mg KLL Auger signal exhibited a kinetic energy of 1177 eV.^36^

Scanning electron microscopy (SEM) of **MicroMg** revealed the formation of well-defined micro cubic–octahedral structures with average dimensions of 1.5 × 2.4 μm (Figure 3a and Figure S2). Scanning transmission electron microscopy (STEM) further confirmed their crystalline nature (Figure 3b). These results highlight the crucial role of the protein scaffold in directing the morphology and structural definition of the biohybrid, demonstrating the successful formation of a microstructured material.

**Figure 3:**
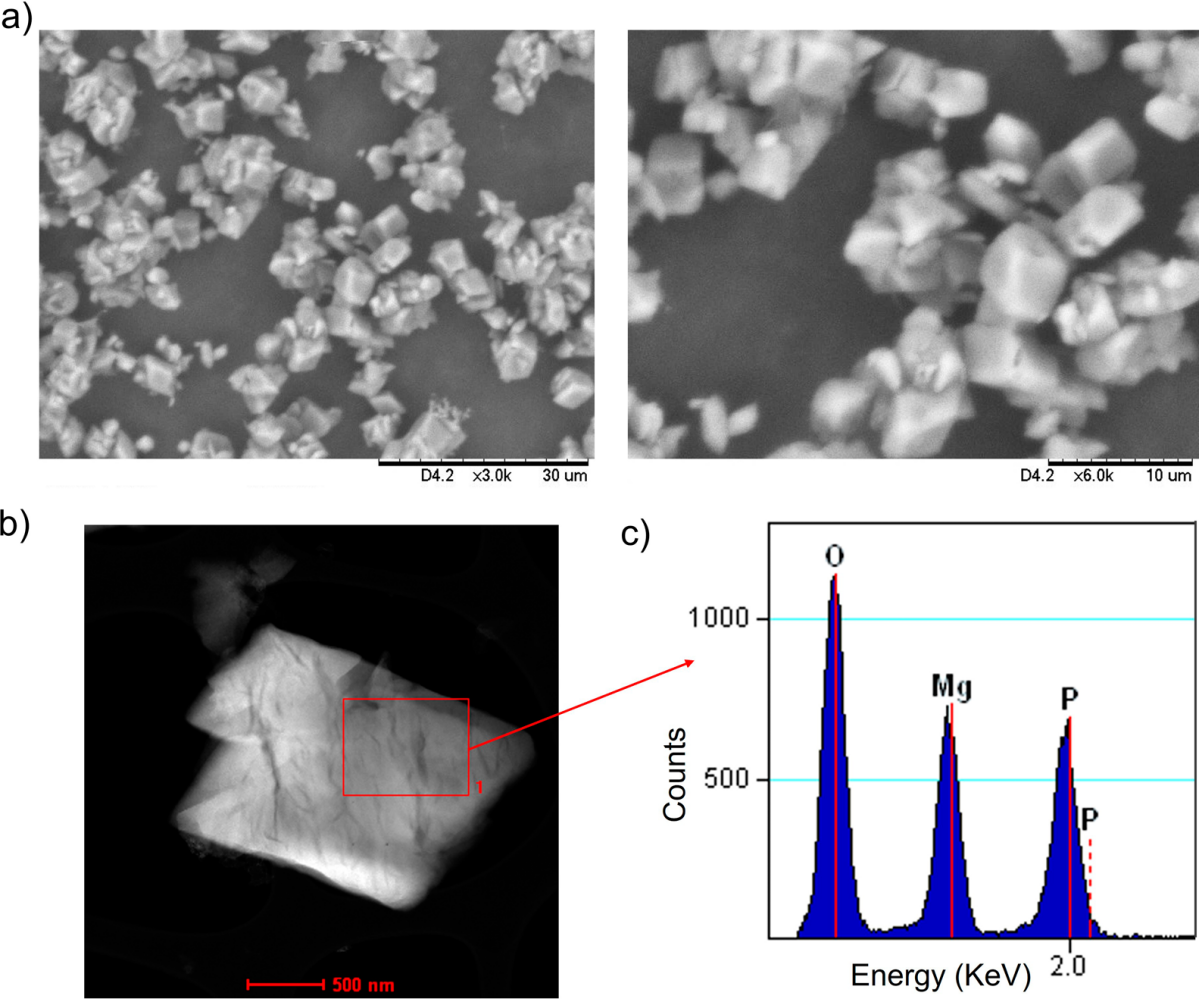
Characterization of **MicroMg**; (a) scanning electron microscopy (SEM) images; (b) scanning transmission electron microscopy (STEM) image; (c) energy-dispersive X-ray spectroscopy (EDX) spectrum.

The elemental composition of the microparticles was analyzed by energy-dispersive X-ray spectroscopy (EDX) (Figure 3c), which revealed the presence of Mg, P, and O, confirming the incorporation of Mg_3_(PO_4_)_2_ in **MicroMg**. Together with the complementary data obtained from XRD, FT-IR, and XPS analyses, these results demonstrated that Mg_3_(PO_4_)_2_ is the sole crystalline phase present in the biohybrid. Finally, inductively coupled plasma–optical emission spectrometry (ICP-OES) analysis indicated that the magnesium content in **MicroMg** was 8%.

### CO_2_ Liquid-Phase Transformation Reaction

Next, the performance of **MicroMg** in the transformation of CO_2_ in aqueous media at room temperature was evaluated under different conditions (Figure S3). The time-dependent evolution of the reaction was first monitored (from 1 min to 2 h). As shown in Figure 4a, the CO_2_ conversion increased progressively up to 30 min, reaching 97% (yielding 265 ppm bicarbonate, 35 ppm formic acid, and 2 ppm methanol), with a turnover frequency (TOF) value of 16 h^–1^. Beyond this point, complete CO_2_ transformation was achieved after 1 and 2 h, with only the distribution of the products slightly changing. In particular, the concentration of formic acid increased by 13% after 2 h, reaching 44 ppm. Therefore, 30 min was selected as the optimal reaction time. These results demonstrate that a complete CO_2_ transformation can be accomplished using this micromaterial. The reaction produced three products: bicarbonate (B) as the major species, followed by formic acid (F) and methanol (M, < 1%), suggesting a sequential conversion from bicarbonate to formic acid and then to methanol.

**Figure 4:**
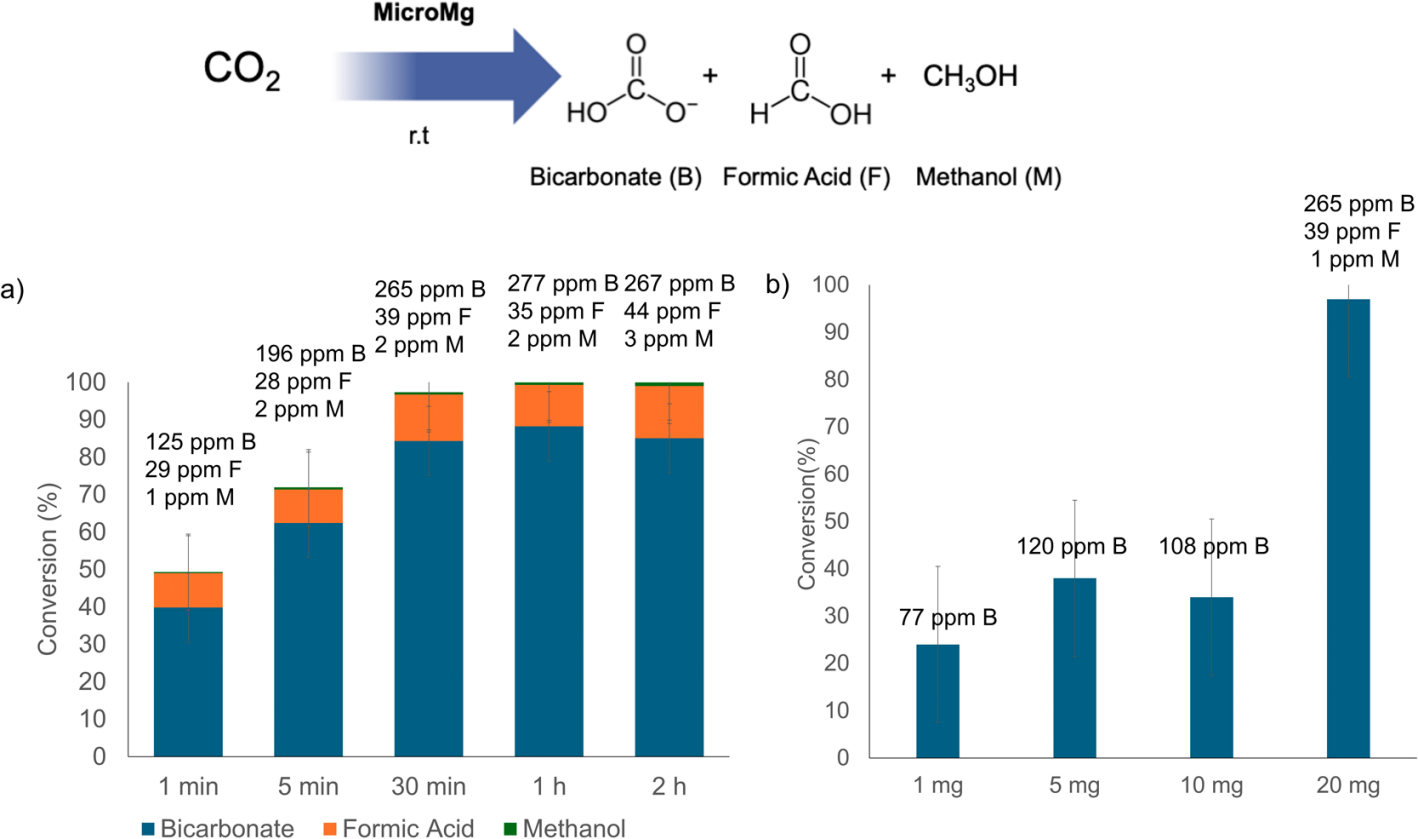
CO_2_ liquid-phase transformation reaction using **MicroMg**; (a) effect of reaction time (aqueous media, room temperature, 20 mg of the catalyst, 314 ppm CO_2_); (b) effect of catalyst loadings (aqueous media 5 mL, room temperature, 30 min reaction time, 314 ppm CO_2_).

Subsequently, the influence of different amounts of the hybrid on the CO_2_ conversion rate was examined (Figure 4b). The best performance was obtained with 20 mg of the catalyst, achieving 97% conversion, whereas the use of only 1 mg led to a drastic decrease of more than 60%. Similarly, 5 and 10 mg resulted in lower conversion percentages, producing solely bicarbonate as the reaction product.

In contrast, with 20 mg of the catalyst, bicarbonate, formic acid, and methanol were obtained. Based on these results, 20 mg was selected as the optimal catalyst loading. Finally, XRD analysis was carried out after the reaction. The diffraction patterns remained unchanged, confirming that the hybrid preserved its structure during the CO_2_ transformation (Figure S4).

Therefore, **MicroMg** proved to be an efficient and stable catalyst for the aqueous transformation of CO_2_ at room temperature, achieving complete conversion within short reaction times and selectively yielding bicarbonate as the main product.

### MicroMg-Paint Coating on Real Wall Surfaces and CO_2_ Gas-Phase Transformation Reaction

With the aim of demonstrating the potential to reduce CO_2_ concentrations in enclosed spaces, this system was applied to coat real wall surfaces. Different paint formulations containing **MicroMg** (the amount ranging from 174 to 700 ppm) were prepared (Table 1 and Figure S5). The resulting mixture was applied as a coating on a 24 cm^2^ section of wall pieces using a brush and then left to dry at room temperature. SEM images confirmed the material adhered to the surface (Figure 5a and Figure S6), whereas paint alone produced a smooth, homogeneous surface. Moreover, it is important to highlight that both the catalyst structure and the chemical integrity of the micromaterial are preserved within the paint, as confirmed by XRD analysis (Figure S4).

**Figure 5:**
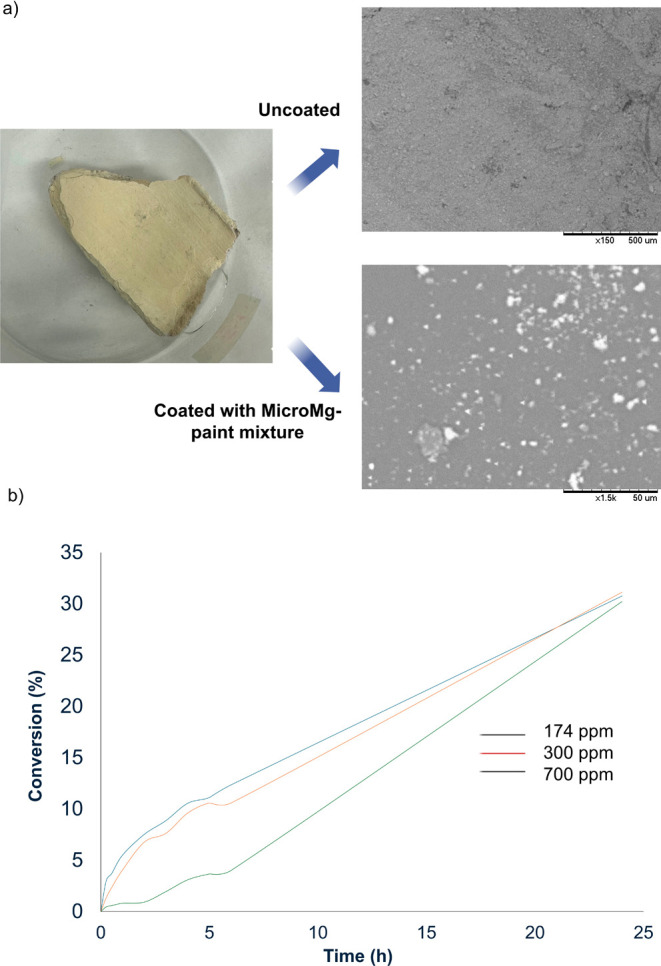
(a) SEM images of uncoated and coated wall piece surfaces (paint containing 174 ppm **MicroMg**); (b) CO_2_ gas-phase transformation using painted wall pieces containing **MicroMg** at different concentrations in ppm.

The gas-phase reaction was then evaluated by placing a wall section in a sealed chamber equipped with a CO_2_ sensor (Figure S7). An initial CO_2_ concentration of 900 ppm was introduced, and a control experiment without wall pieces was performed to assess the stability of the system. CO_2_ levels were monitored over 6 h (Figure S8), showing a loss of less than 2%, which confirms the durability of the sealed setup. Figure 5b shows the CO_2_ conversion profiles over 24 h for the different wall pieces with different **MicroMg** concentrations. After 24 h of incubation, the three painted wall pieces containing **MicroMg** (174, 350, and 700 ppm) reached a similar overall conversion (∼30%) (Figure 5b); however, great differences were observed for example at 6 h of incubation, where 12% CO_2_ was reduced with the piece containing 174 ppm **MicroMg**, whereas a CO_2_ reduction of 10% or even 4% was observed at higher concentrations (pieces with 350 and 700 ppm **MicroMg**, respectively) (Figure 5b).

These results indicated that a low **MicroMg** concentration was sufficient for effective CO_2_ transformation, and this paint formulation (containing 174 ppm) was selected for further experiments.

### Evaluation of Washing Cycles of MicroMg-Paint Coating on Real Wall Surfaces

The effect of washing cycles on the wall piece coated with the painted formulation with 174 ppM **MicroMg** was evaluated. After the first CO_2_ transformation following the procedure described above, the wall piece was washed with distilled water and left to dry at room temperature. Then, this piece was introduced into the chamber for repeating the CO_2_ transformation reaction. This procedure was performed for a total of three washing cycles. The reaction profiles obtained with the fresh catalyst and after three cycles were very similar (Figure 6a). In fact, after 24 h of reaction and two washing cycles, a conversion of around 30% was achieved, which is comparable to the initial value. After the third cycle, the coating maintained almost 90% of its efficiency.

**Figure 6:**
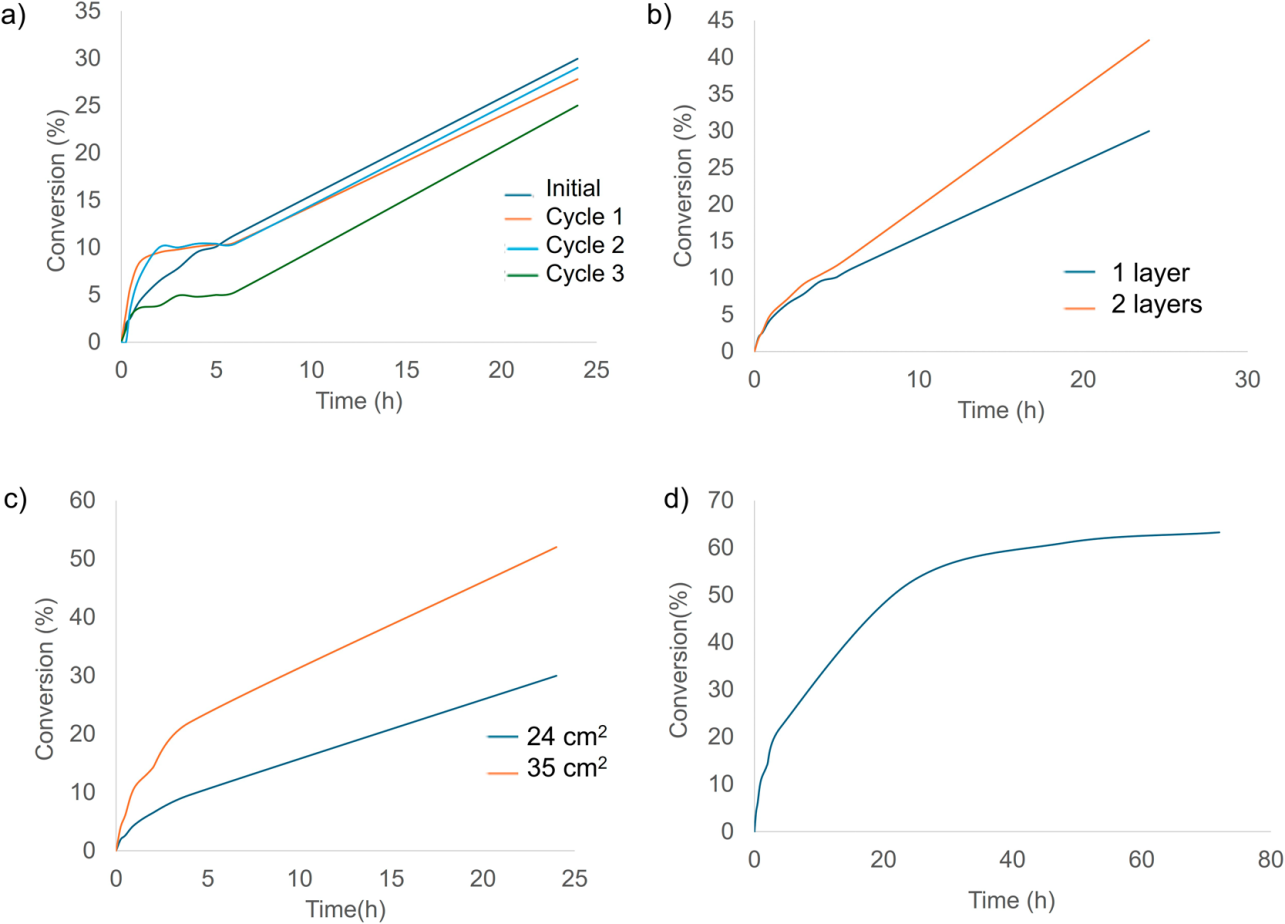
CO_2_ gas-phase transformation on the wall piece coated with paint containing the **MicroMg**-174 ppm. (a) Evaluation of washing cycles; (c) effect of the coated surface area; (b) effect of layers; (d) effect at higher CO_2_ concentrations.

### Evaluation of the Effect of the Coated Surface Area

The impact of applying the MicroMg-paint mixture over a larger surface area was evaluated. In this case, the reaction was carried out on a wall piece of 35 cm^2^ and compared with results obtained using a 24 cm^2^ one (Figure 6c). A larger coated surface resulted in a higher CO_2_ transformation, reaching 50% conversion—almost double that obtained with 24 cm^2^ (30%)—with a corresponding transformation rate of 30 ppm CO_2_/h. These results indicate that increasing the catalytic surface significantly enhances the CO_2_ conversion in the same chamber with the same CO_2_ concentration.

### CO_2_ Gas-Phase Transformation Using a Double MicroMg-Paint Layer

To further enhance CO_2_ conversion, the effect of applying a second paint layer to the painted wall piece was studied. The same procedure of coating the wall piece described above was performed now using an already painted wall piece, introducing an additional layer. Then, the piece was left drying at room temperature. Then, this new double coated piece was tested in the reaction. After 24 h of reaction, the reduction of CO_2_ concentration in the chamber increased to 42%, compared to 30% obtained with a single layer (Figure 6b), with a transformation rate of 16 ppm CO_2_/h, nearly twice that observed with one layer. This demonstrates that applying an additional paint layer improves the catalytic performance, maybe because of the methodology applied (brush); a second application allowed a full coating of the wall surface.

### CO_2_ Gas-Phase Transformation at Higher CO_2_ Concentrations

Finally, the catalytic activity of **MicroMg**-paint in a wall piece of 24 cm^2^ was evaluated under higher CO_2_ concentrations inside the reactor chamber. A concentration of 1500 ppm of CO_2_—almost double the standard amount—was introduced. The reaction was monitored for 72 h due to the higher initial concentration (Figure 6d). After 24 h, a conversion of 52% was achieved, which increased to 61% at 48 h and stabilized at an approximately 63% CO_2_ transformation after 72 h. These findings indicated that longer reaction times lead to higher CO_2_ conversion and that **MicroMg** is capable of effectively transforming elevated CO_2_ concentrations (up to 1500 ppm), demonstrating its potential for reducing CO_2_ in indoor environments with high gas levels. The results at 72 h seem to indicate that saturation of the surface was achieved, probably by products formed. Then, after washing the surface of the wall piece, this was applied again in the reaction, obtaining similar results (data not shown).

### Proposed Mechanism for the Reduction of CO_2_ to Bicarbonate, Formic Acid, and Methanol

The reduction of CO_2_ to value-added products using **MicroMg** proceeds through a stepwise mechanism in aqueous, CO_2_-saturated media (Figure 7). In this system, magnesium phosphate nanoparticles are stabilized by an enzyme scaffold via coordination to aspartate and glutamate residues, which not only anchor the nanoparticles but also participate in the proton transfer and stabilization of reaction intermediates.

**Figure 7:**
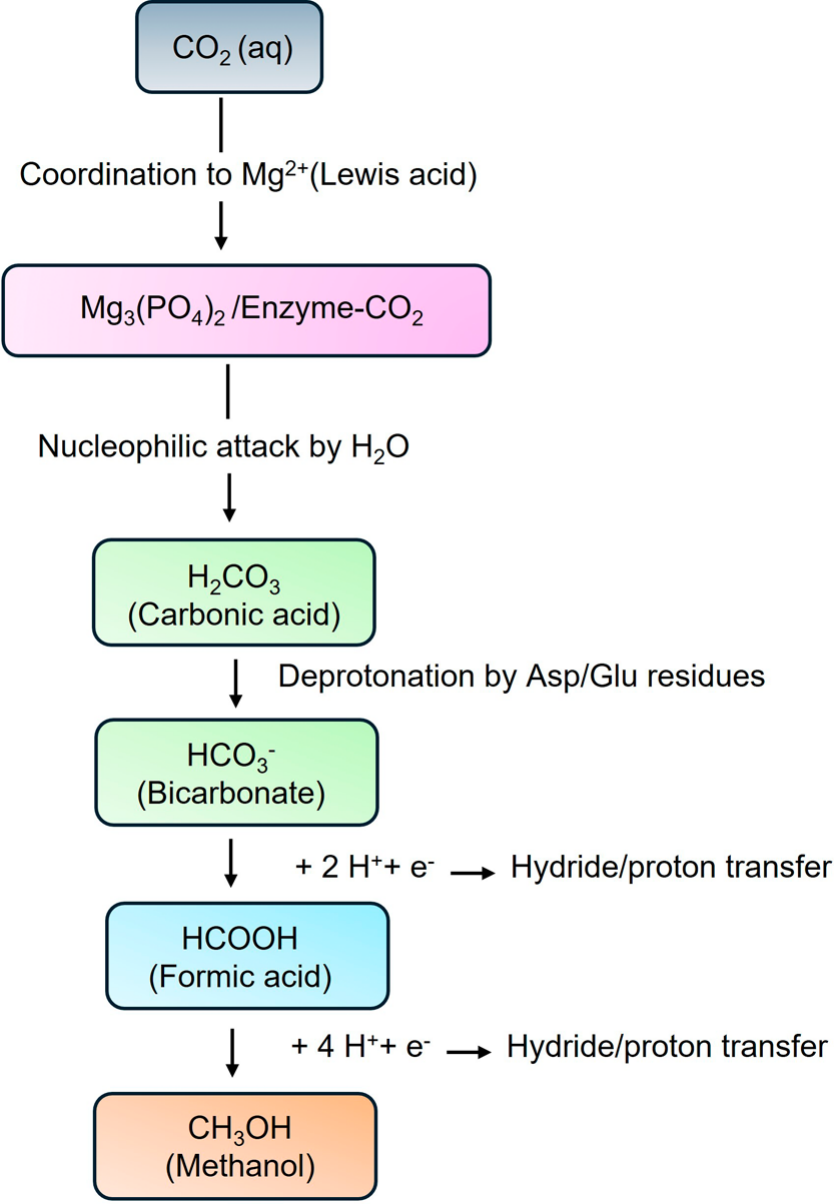
Proposed mechanism for the reduction of CO_2_ to bicarbonate, formic acid, and methanol using MicroMg.

In the first step, CO_2_ is activated through coordination to the Lewis acidic Mg^2+^ sites within the phosphate lattice. This coordination polarizes the carbon–oxygen double bond, rendering the carbon atom more electrophilic and susceptible to nucleophilic attack. Water molecules in the aqueous medium subsequently attack activated carbon, forming carbonic acid as an intermediate. Proton transfer, facilitated by nearby phosphate groups and the enzyme residues, then leads to the formation of bicarbonate (HCO_3_^–^), which is stabilized at the catalyst surface. This step represents the initial conversion of CO_2_ to an activated, reactive species suitable for reduction.^37^ The second step involves the reduction of bicarbonate to formic acid (HCOOH). Bicarbonate binds to a Mg^2+^ site on the catalyst surface, further activating the carbon center. Hydride transfer from water, a photochemical source, or another external reducing agent attacks the activated carbon, while protonation from the medium or enzyme residues stabilizes the intermediate, producing formic acid.^37^ The combined action of Mg^2+^ coordination, phosphate-mediated proton shuttling, and enzyme stabilization ensures efficient conversion, while maintaining high selectivity.

Finally, formic acid undergoes sequential reduction to methanol (CH_3_OH). Formic acid binds to the Lewis acidic Mg^2+^ site, where successive hydride and proton transfers reduce the carbon center to methanol.^38^ Throughout this process, phosphate groups and enzyme residues facilitate proton shuttling, stabilize reactive intermediates, and maintain the proximity between active sites and substrate molecules. The overall reaction sequence can thus be summarized as CO_2_ → HCO_3_^–^ → HCOOH → CH_3_OH, with **MicroMg** providing both structural support and catalytic activation.

This mechanistic framework highlights the dual role of the catalyst: magnesium phosphate nanoparticles serve as Lewis acid centers for substrate activation, while the enzyme scaffold stabilizes the nanoparticles, facilitates proton transfer, and ensures efficient interaction between the substrate and active sites. The proposed stepwise mechanism provides a rational explanation for the observed formation of formic acid and methanol under aqueous CO_2_-saturated conditions.

## Conclusions

In this work, we have developed a magnesium-based biohybrid, MicroMg, which forms a microstructured cubic–octahedral material composed of magnesium phosphate. MicroMg efficiently transforms CO_2_ into bicarbonate under ambient conditions. When applied to real wall surfaces, it retained catalytic activity over three washing cycles, maintaining around 90% of its initial efficiency. Increasing the coated surface area from 24 to 35 cm^2^ nearly doubled CO_2_ conversion, while applying a second layer of MicroMg-paint roughly doubled the transformation rate compared to a single layer. Additionally, **MicroMg** remained highly active at elevated CO_2_ concentrations (up to 1500 ppm), achieving over 60% conversion after 72 h, with a transformation rate of 16 ppm CO_2_/h in practical atmospheric CO_2_ mitigation. These results demonstrate that MicroMg is a reusable, high-performing, and scalable material for greenhouse gas mitigation, providing an effective and practical strategy for indoor CO_2_ reduction.

Future applications may include the integration of MicroMg-based coatings into architectural surfaces, smart building materials, and air management systems, enabling continuous CO_2_ mitigation in indoor environments. Further development toward large-scale coating technologies, durability under long-term operation, and compatibility with existing construction materials will be essential to translating this approach into real-world sustainable building solutions.

## Supplementary Material



## Notes

Synthetic procedure and characterization of **MicroMg** (Figures S1 and S2) and synthetic procedure of the CO_2_ reaction (Figures S3–S9) (PDF)
