# Supplementary material for: Design of a Magnesium Microstructured Biohybrid Material for Practical Atmospheric CO2 Mitigation
Source: ACS Appl Energy Mater. 2026 Feb 10;9(4):2248–56. doi: 10.1021/acsaem.5c03841 (PMC12933514; doi:10.1021/acsaem.5c03841)
Supplement: Supplementary file 1 [file ae5c03841_si_001.pdf]

Supporting information

Design of a Magnesium Microstructurated Biohybrid Material for Practical Atmospheric CO_2 Mitigation

Carla Garcia-Sanz[1] and Jose M. Palomo*, [1]

[1] Instituto de Catálisis y Petroleoquímica (ICP), CSIC, c/Marie Curie 2, Campus UAM Cantoblanco, 28049 Madrid (Spain)

*Correspondence: josempalomo@icp.csic.es

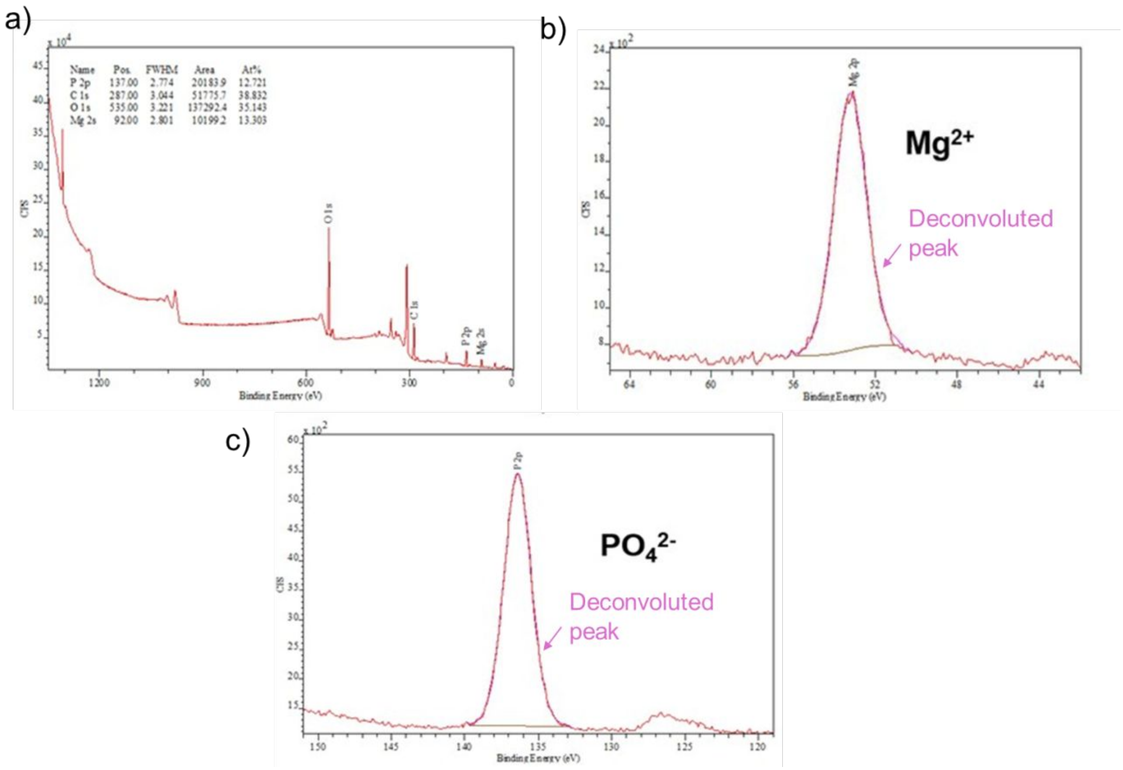

Figure S1. X-ray photoelectron spectroscopy (XPS) characterization of **MicroMg**: a) survey spectrum; b) P2p spectrum; c) Mg2p spectrum.

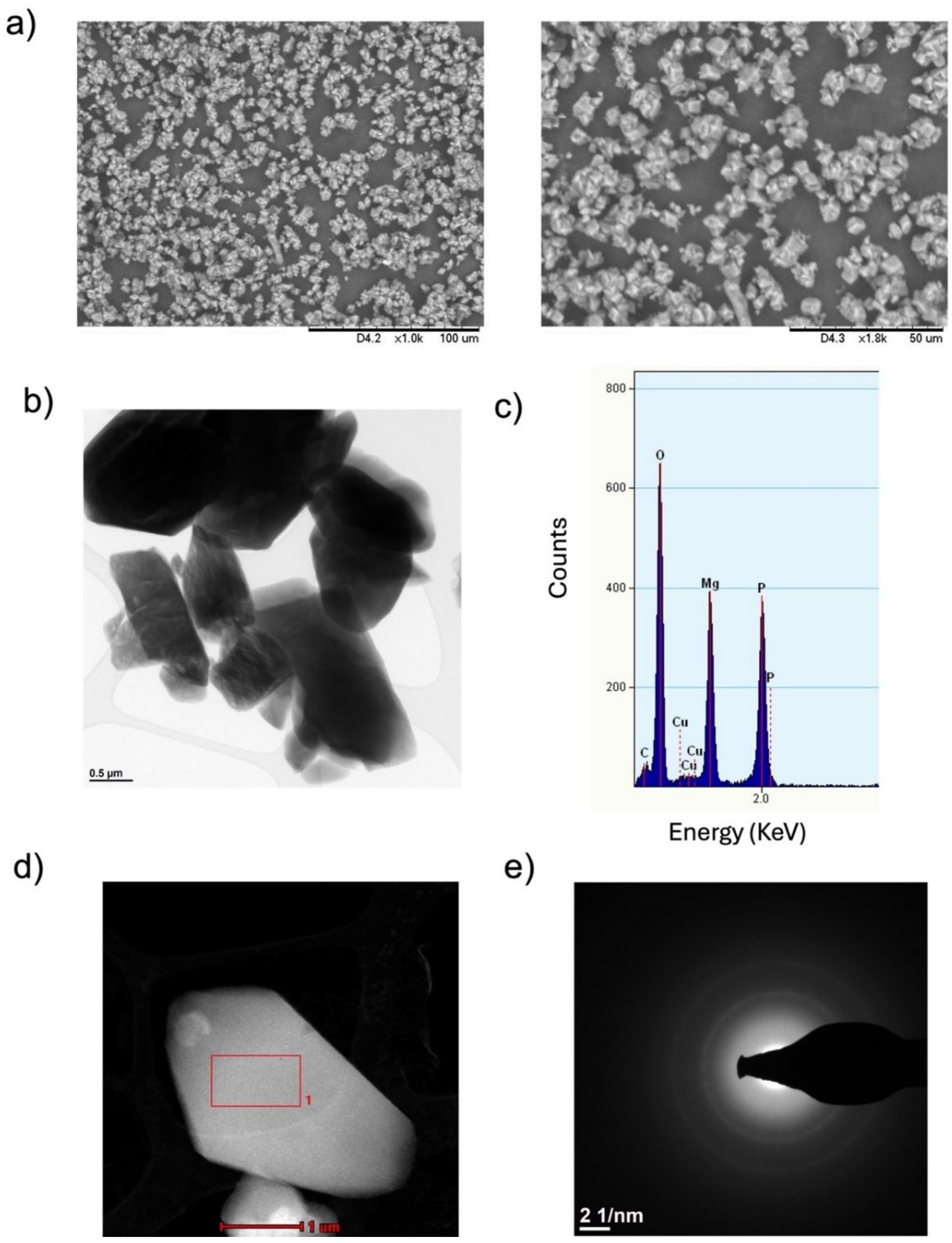

Figure S2. Characterization of **MicroMg**. a) Scanning electron microscopy (SEM). b) Transmission electron microscopy (TEM). c) Energy Dispersive X-ray Spectroscopy (EDX) spectrum. *d)* High-Angle Annular Dark-Field Scanning Transmission Electron Microscopy (HAADF-STEM) image and e*)* SAED image.

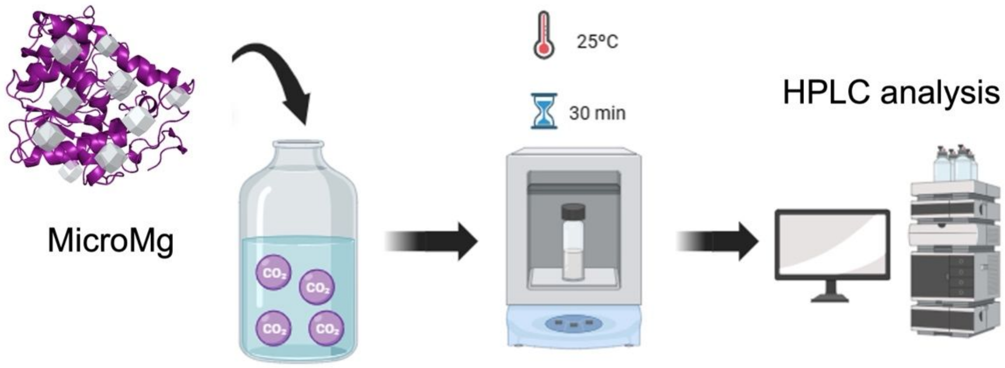

Figure S3. Schematic representation of the CO_2 liquid-phase transformation reaction using MicroMg. Step 1: addition of MicroMg to an aqueous solution saturated with 314 ppm CO_2. Step 2: incubation of the mixture at room temperature for 30 minutes. Step 3: analysis of the reaction products by HPLC.

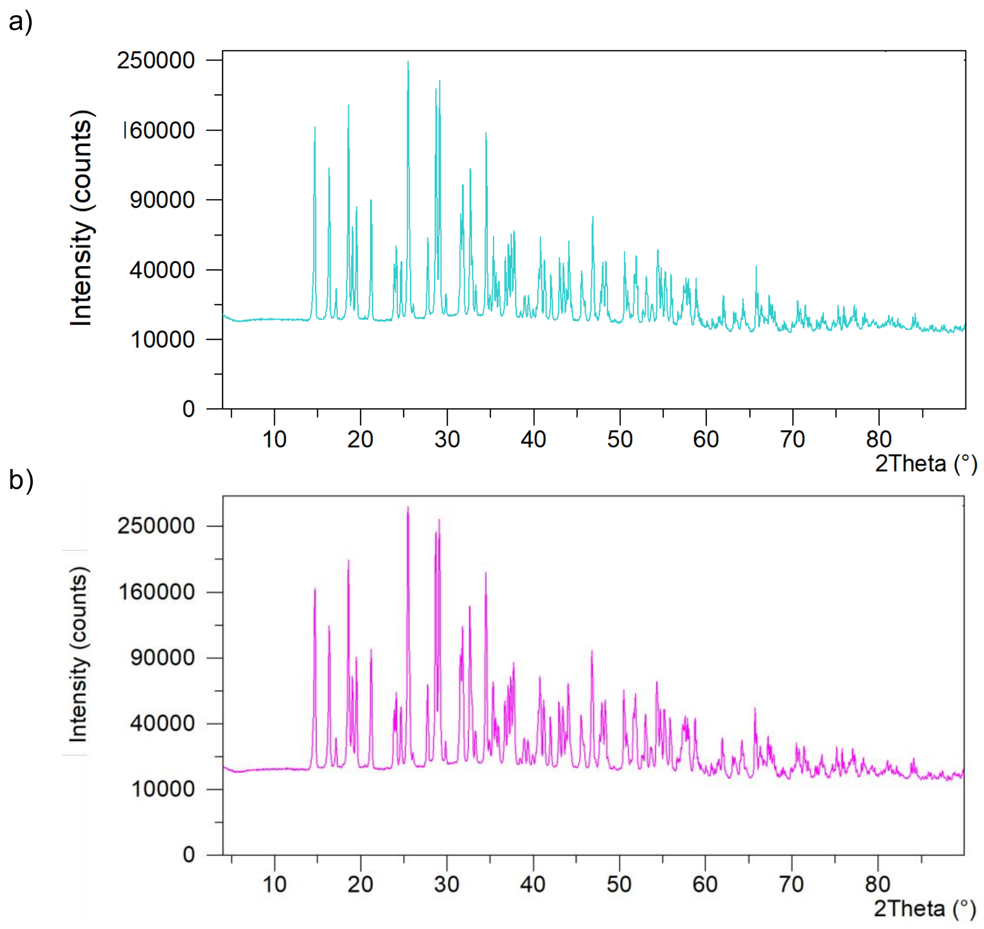

Figure S4. XDR pattern of **MicroMg** (a) and after the CO_2 transformation reaction (b).

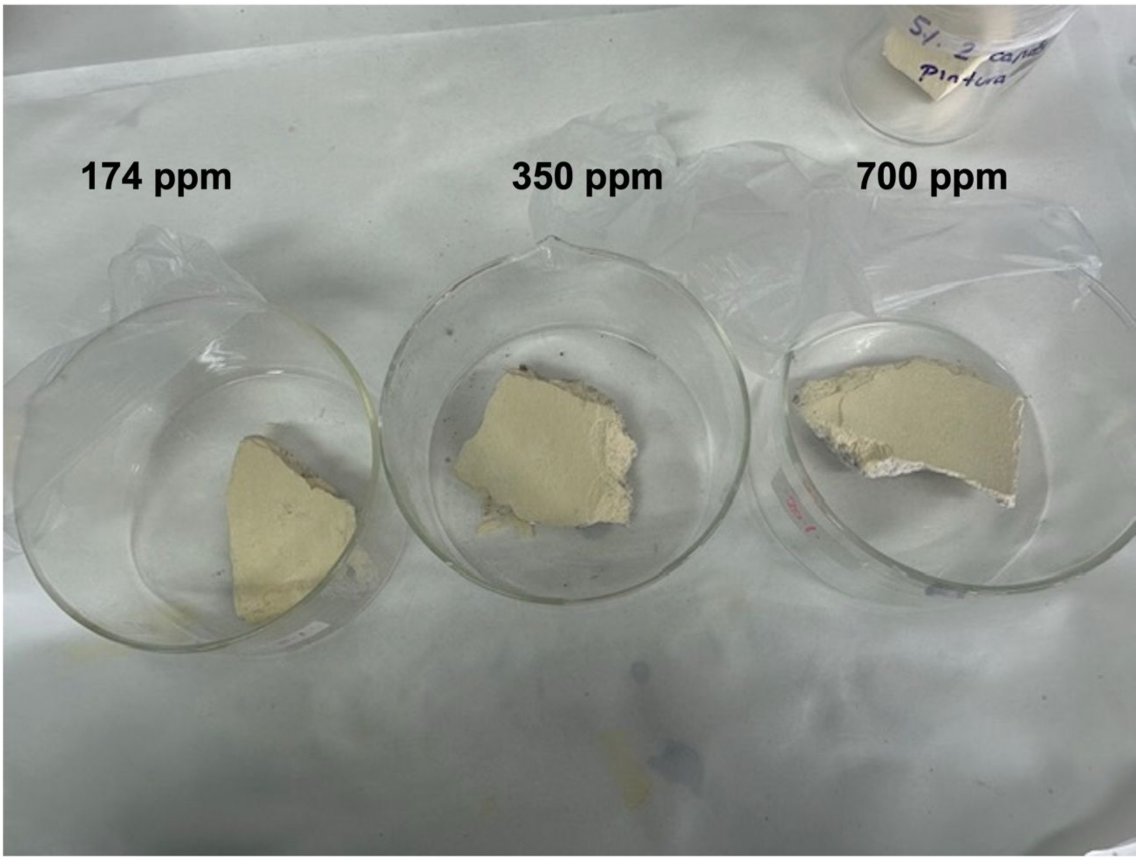

Figure S5. Real wall surfaces coated with the MicroMg–paint mixture at different MicroMg concentrations.

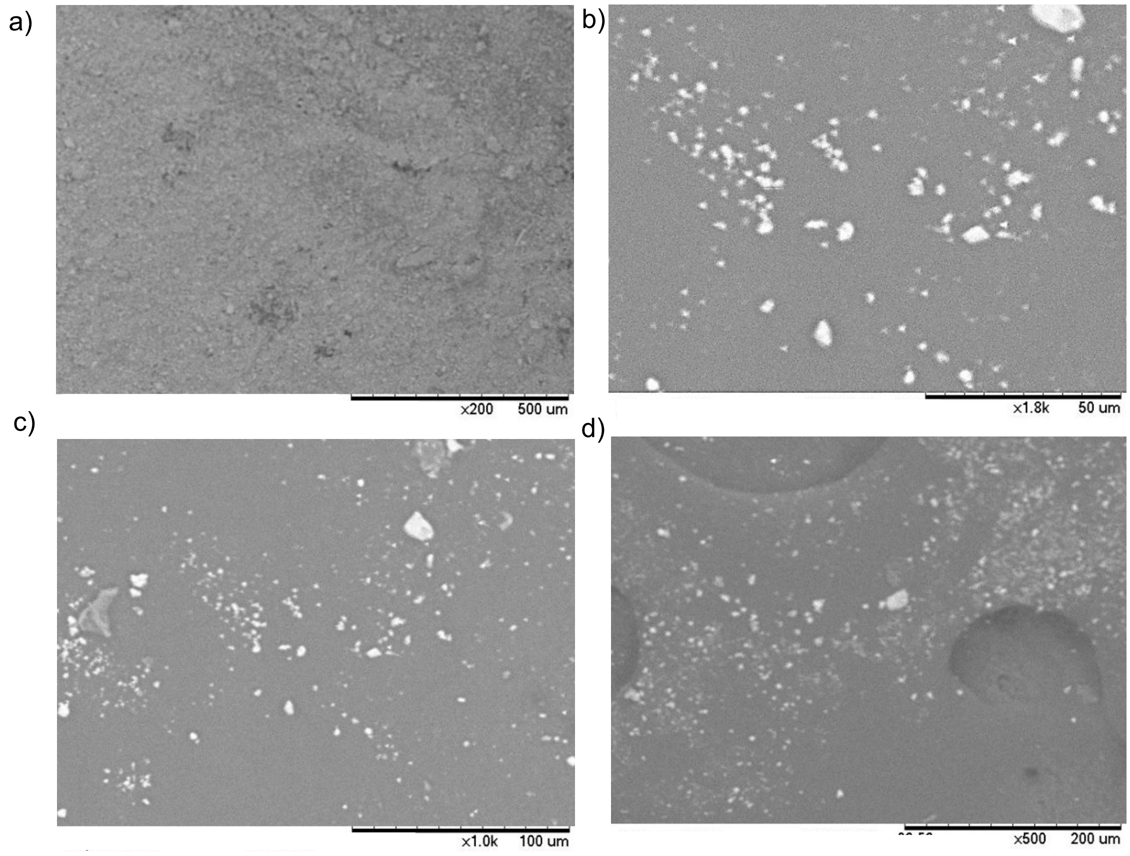

Figure S6. SEM images of real wall surfaces coated with the MicroMg–paint mixture at different MicroMg concentrations: a) uncoated wall; b) 174 ppm; c) 350 ppm; d) 700 ppm.

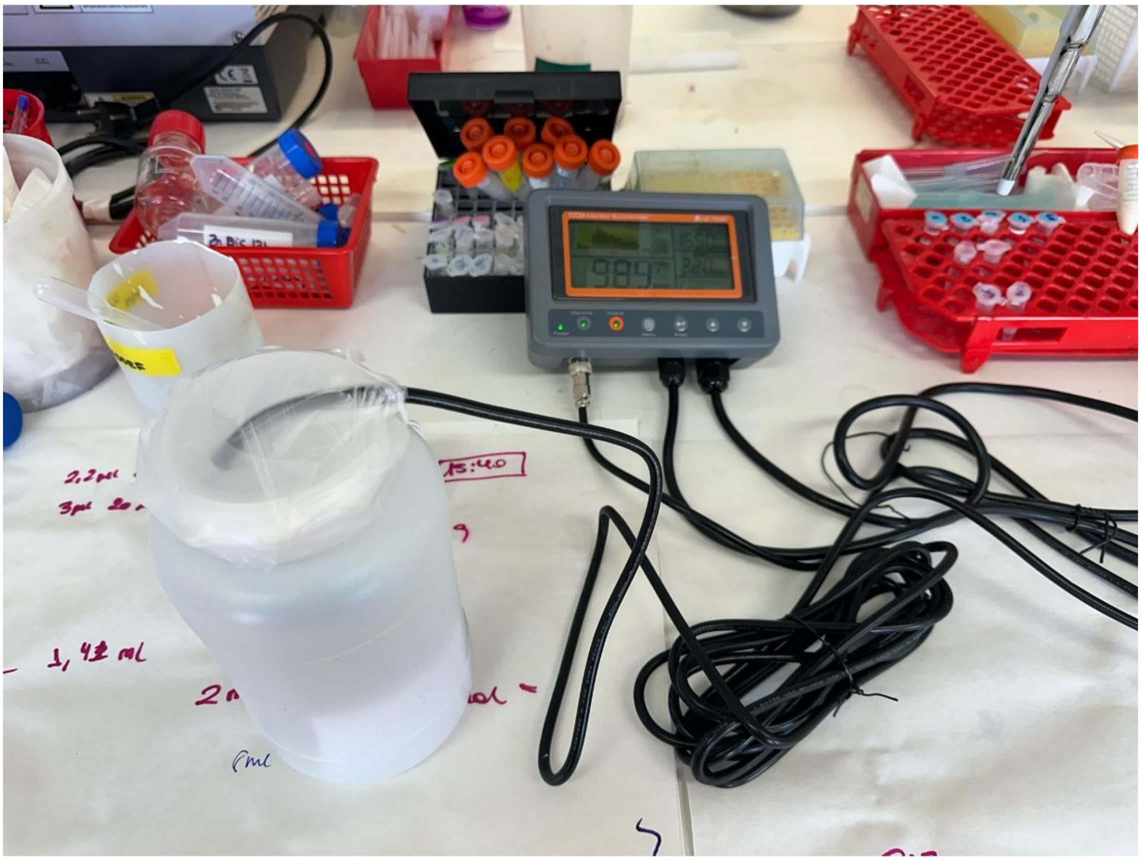

Figure S7. Sealed chamber equipped with a CO_2 sensor used for the CO_2 gas-phase transformation experiments.

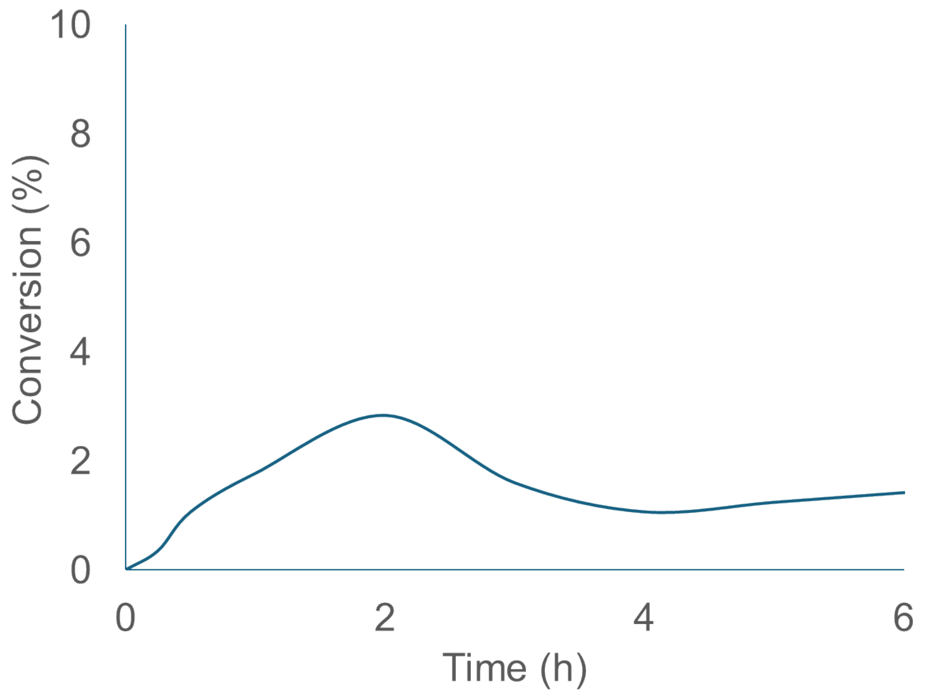

Figure S8. Stability of the sealed system over 6 h, showing the monitored CO_2 levels during the gas-phase transformation reaction.
